# A Non-Destructive, Tuneable Method to Isolate Live Cells for High-Speed AFM Analysis

**DOI:** 10.3390/microorganisms9040680

**Published:** 2021-03-25

**Authors:** Christopher T. Evans, Sara J. Baldock, John G. Hardy, Oliver Payton, Loren Picco, Michael J. Allen

**Affiliations:** 1 Plymouth Marine Laboratory, Plymouth PL1 3DH, UK; chev@pml.ac.uk; 2 Interface Analysis Centre, Wills Physics Laboratory, University of Bristol, Bristol BS8 1TL, UK; oliver.payton@bristol.ac.uk; 3 Department of Chemistry, Lancaster University, Lancaster LA1 4YB, UK; s.baldock@lancaster.ac.uk (S.J.B.); j.g.hardy@lancaster.ac.uk (J.G.H.); 4 Materials Science Institute, Lancaster University, Lancaster LA1 4YB, UK; 5 Department of Physics, Virginia Commonwealth University, Richmond, VA 23284, USA; lpicco@vcu.edu; 6 Department of Biosciences, College of Life and Environmental Sciences, University of Exeter, Stocker Road EX4 4QD, UK

**Keywords:** high-speed, atomic force microscopy, microalgae, microbe, immobilization, multiphoton polymerization, 3D printing

## Abstract

Suitable immobilisation of microorganisms and single cells is key for high-resolution topographical imaging and study of mechanical properties with atomic force microscopy (AFM) under physiologically relevant conditions. Sample preparation techniques must be able to withstand the forces exerted by the Z range-limited cantilever tip, and not negatively affect the sample surface for data acquisition. Here, we describe an inherently flexible methodology, utilising the high-resolution three-dimensional based printing technique of multiphoton polymerisation to rapidly generate bespoke arrays for cellular AFM analysis. As an example, we present data collected from live *Emiliania huxleyi* cells, unicellular microalgae, imaged by contact mode High-Speed Atomic Force Microscopy (HS-AFM), including one cell that was imaged continuously for over 90 min.

## 1. Introduction

Atomic force microscopy (AFM) has been providing insight into nanoscale features, events and processes since its development in 1986 [1]. AFM is of particular suitability for biological structure-function relationship elucidation, with the ability to measure and investigate aspects of interest, including sample surface topography and mechanical properties, under physiologically relevant conditions [2].

A challenging feature of biological sample preparation for high-resolution AFM imaging, in air or liquid environments, is suitable immobilisation on to solid substrates. Adhesion has to withstand forces exerted by the AFM tip throughout data acquisition which can deform or even displace soft samples during imaging. The diameter of a biological cell compared to the height of the tip is often comparable, consequently, there is danger that the tip cannot effectively traverse the cell height differential and instead dislodges the sample.

Indeed, animal cells and other eukaryotes could be considered the antithesis of model AFM samples. Their structural softness, roughness, size and height variance and potential low contact area (e.g., spherical cell under minimally applied vertical tip forces) to a flat substrate (typically used for AFM imaging) offer challenging sample preparation issues. A well prepared, immobilized sample allows for high-resolution imaging, without modifying or distorting the structure of interest. With this in mind, several methods have been developed to address these preparation issues [3].

Depending on cell shape, occasionally a simple preparation of air drying onto mica or direct centrifugation onto mica under aqueous conditions [4] may be possible. Microorganisms such as bacteria can be directly chemically fixed to a smooth substrate. Gelatin and poly-L-lysine coated mica surfaces have been shown to immobilise Gram positive and negative samples to differing efficiencies [5]. Other cell types such as diatoms have also been found to adhere to mica coated with poly-L-lysine [6,7] or polyethylenimine [8,9]. A differing approach is to physically immerse the sample in a solidifying matrix such as agar [10]. Cyanobacteria partially imbedded in dental wax have also been suitably immobilised for AFM analysis [11]. Previously, we have utilised an aluminium hydroxide derived hydrogel matrix for imaging imbedded spherical *C. sorokiniana* cells [12]. A drawback to these fixations and immersions is the possibility of altered biological surfaces, cantilever tip pollution, uneven background and difficulty controlling background heights.

Filtering and trapping spherical shaped cells in track etched polycarbonate membranes with pore sizes comparable to cell diameter is an extremely straight forward procedure [13]. This allows an elegant mechanical isolation of the cell with minimal sample alteration which simultaneously minimizes the Z axis height differential. However, in practice, cells fill pores with low success rate, pores are time consuming to locate due to their random arrangement and cell height within the pore is uncontrolled with generic poorly defined pore depth. Identifying suitable cells for high definition analysis is difficult with these membranes. Immobilising single cells with a patch clamp micropipette circumvents this difficulty [14,15], but requires additional instrumentation setup challenges and only one sample cell can be imaged at a time. The most recent improvement to the foundation polycarbonate membrane method involves trapping cells in micro structured polydimethylsiloxane (PDMS) stamps by capillary deposition [16]. This method is a controlled way of preparing and gathering statistically significant data from multiple cells. PDMS stamps require multiple fabrication steps in the creation of a microstructured silicon master (~1 week for fabrication) and PDMS stamp moulding (~3 h) with stages requiring degassing under vacuum and crucial, manual cutting and demoulding of the microstructured PDMS motif.

The ideal solution is a regular array, for ease of sample location, with flexible shape and size design for differing samples. The array should be non-toxic, have a low and regular background, be cheap and fast to produce and be able to support multiple live cells simultaneously under optimal conditions. Here, we present a method for preparing live cells for contact mode high-speed atomic force microscopy (HS-AFM); utilising the versatility of multiphoton polymerisation (Nanoscribe direct laser writing) to 3D print bespoke designed arrays to study our cell strains of interest.

## 2. Materials and Methods

### 2.1. Array Design

Designs were created in AutoCAD (AutoDesk, San Rafael, CA, USA) and imported into the Nanoscribe Photonic Professional GT scripting program DeScribe 2.4 (NanoScribe GmbH, Karlsruhe, Germany) as three-dimensional (3D) stereolithography format (.stl) files. 3D structures were transformed into xyz commands using a Z slicing of 0.3 micron and 200 nm hatching, and compiled into a command script with the laser writing speed and power. Structures were processed to ensure sufficient overlap of the polymerised voxels (Z-slicing and X&Y hatch settings) for structural integrity.

### 2.2. Array Production

A Photonic Professional GT (Nanoscribe GmbH) equipped with a 100 fs pulsed 780 nm laser focussed with microscale ×63 (Numerical aperture 1.4) laser objective was used. Direct Laser Writing (DLW) was carried out using ‘conventional’ mode. Index matched oil was coupled to a methacrylate coated glass coverslip with a dropcast resist (Nanoscribe IP-L, Nanoscribe GmbH) on top. 38% laser power (~16 mW) at 20,000 µm/s was used. Post DLW the non-polymerised monomer solution was removed by immersing the coverslips in a developer solution, propylene glycol methyl ether acetate (PGMEA) for 20 min, followed by an isopropyl alcohol (IPA) rinse for 5 min. The slides were blown dry with nitrogen.

Methacrylation of slides was carried out using a 3 stage: clean, activate and modify process. This was adapted from [17]. Coverslips were immersed for 30 min in 1 M NaOH, rinsed in deionised water, immersed for 30 min in 1 M HCl, rinsed in deionised water, dried in a stream of nitrogen. Then, 200 µL of 20 *v*/*v* % 3-trimethyloxysilyl propyl methacrylate solution prepared in ethanol was added to each coverslip and allowed to evaporate before second application. After 30 min they were then dipped in acetone and dried in a stream of nitrogen.

### 2.3. Algal Cell Culture

Non-calcifying *Emiliania huxleyi* (*E. huxleyi*) cells (CCMP 374, National Culture Collection of Marine Phytoplankton for the USA, Bigelow Laboratory for Ocean Sciences, Boothbay, ME, USA) were maintained at 17 °C under a 16:8 h light:dark cycle in f/2 media [18] (enriched Atlantic seawater, salinity ~33 ppt determined by refractometer) in a Versatile Environmental Test Chamber (Sanyo MLR-350, Osaka, Japan).

### 2.4. Optical/Fluorescent Microscopy

Optical and fluorescent microscopy was performed on a Lecia DM IL LED Fluo invert microscope with an attached Lecia EL600 compact light source (Lecia Microsystems, UK). The filter to visualise natural fluorescence was an N2.1 small filter. The data was collected and processed using Lecia Application Suite (Las X, 2.0, Lecia Microsystems, UK). Suspected viable cells were located by grid coordinate of filled well with optical and fluorescence microscopy.

### 2.5. Scanning Electron Microscopy

Empty cell capture arrays were analysed under vacuum by Scanning Electron Microscopy (SEM) performed on a JEOL JSM-7800F (JEOL Ltd., Tokyo, Japan) without coating or application of stage bias. Working distance = 9.5 mm, accelerating voltage = 1 kV, magnification = ×500.

### 2.6. HS-AFM

The instrument used was a Bristol Nano Dynamics Ltd. (BND, Bristol, UK) HS-AFM operating in a sample scanned contact mode. The sample is raster scanned using a custom flexure stage over a 5 µm range in X and Y at 1000 Hz (fast) and 1–4 Hz (slow), respectively. The HS-AFM measured the vertical displacement of a low spring constant triangle cantilever (Bruker Nano, MSNL, 0.01 N m^−1^ spring constant, Bruker AFM Probes, CA, USA) at a rate of 2 MS/s. Height data was collated into images (1000 × 1000 pixels) at a rate of 2 fps using BND’s collection software.

For cell capture into fabricated arrays, 100 µL of *E. huxleyi* cells ≥1.2 × 10^5^ mL^−1^ were allowed to gravity settle for 45 min into wells. Slides were then washed with 3 × 1 mL 0.1 M phosphate buffer. 100 µL of buffer was loaded as a grid covering droplet for liquid HS-AFM imaging. Data was collected as video files using the BND software. These could be viewed in further custom HS-AFM Display readback software and individual frames can be exported into .gsf files for further processing and analysis with Gwyddion, an open-source SPM image analysis suite (http://gwyddion.net). Cell membrane data has been processed with scar removal, polynomial alignment of rows and gaussian continuous wavelet transformation for presentation.

## 3. Results

The multiphoton fabrication process (also known as direct laser writing (DLW)) was reviewed in [19], and is a form of high-resolution, three-dimensional (3D) printing. Multi-photon polymerisation occurs when there is simultaneous adsorption of two or more photons to excite a molecule from one energy state to a higher state. These photons can be of identical or differing frequencies, usually excited from the ground state, and are most efficiently produced by pulsed lasers at high intensities. A photoinitiator absorbs the laser light to produce an active species which causes directed polymerisation of the monomer to form a 3D structure. The fabrication method achieves highly localised polymerisation and cross-linking of photopolymers with a tightly focussed femtosecond pulsed laser. This allows for printing of structures with 0.1 µm resolution [20] and true 3D patterning as polymerisation is not limited to the surface of the monomer. This is in contrast to conventional stereolithography which can suffer from high surface tension leading to possible distortion. Other advantages include no requirement for printing supports and a concomitant reduction in oxygen inhibition.

**Figure 1:**
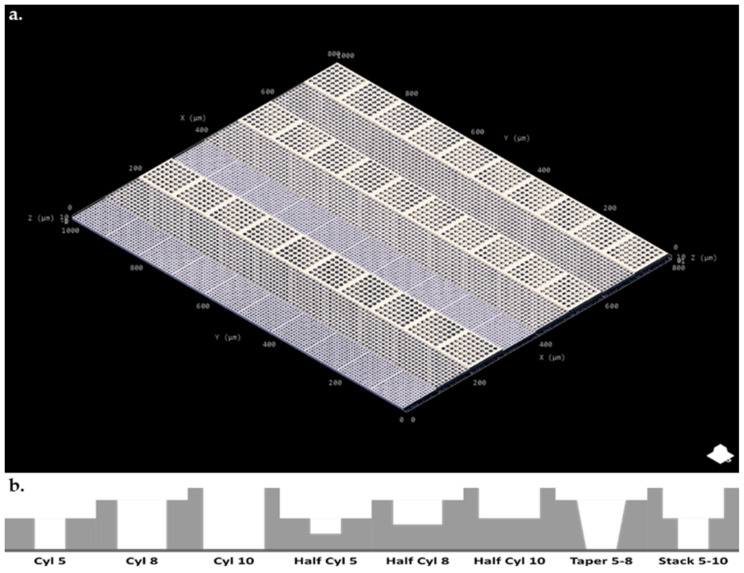
(**a**). AutoCAD Nanoscribe cell array design (**b**). Cross sectional diagram of individual wells seen in each block type.

The multiphoton fabrication ‘3D printing’ method was used to fabricate cell capture arrays featuring wells of 5, 8, 10 µm diameter and 2.5/5, 4/8, 5/10 µm depth, respectively (Figure 1 and Table 1). Design with AutoCAD allowed for quick iterative design (i.e., rapid prototyping) with variations in block and well design and layout easily combined into one single large structure giving intra array versatility and optimization opportunities for specific cell types and morphologies.

**Figure 2:**
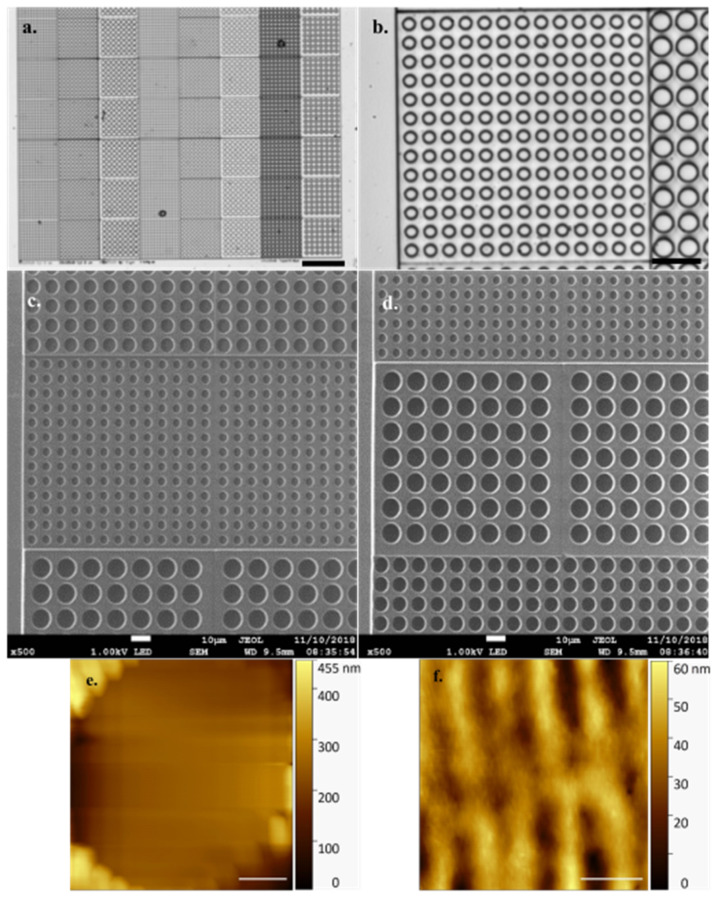
(**a**). Optical microscope images of empty array ×10 obj (Scale bar 100 µm) and (**b**). ×63 obj (Cyl 5) (Scale bar 20 µm) (**c**). Scanning electron micrograph of empty array featuring (scale bars 10 µm) Cyl 8 (top), Cyl 5 (middle), Cyl 10 (bottom) and (**d**). Cyl 5 (top), Cyl 10 (middle), Cyl 8 (bottom) (**e**) Array individual empty cell well (Cyl 5) visualised by high speed-atomic force microscopy (HS-AFM) in liquid (Scale bar 1 µm, Data captured at 2 fps) (**f**) Topography of surface structure generated by Nanoscribe direct laser writing (DLW) method visualised by HS-AFM in liquid (Scale bar 500 nm, Data captured at 2 fps).

Figure 2 shows the precision with which the array is printed during a successful job with no defects. AFM offers high resolution in a liquid environment, giving insight into the pattern and structure of the DLW method’s linear printing regime (Figure 2f) with individual, regular periodicity, print rows (~0.4 µm width/periodicity, ~30 nm measured height) clearly visible in both forming the well and in connecting layers. Exhibiting a refractive index of 1.48, the fabricated arrays are well suited to both optical and fluorescence microscopy, allowing visualisation and identification of the naturally fluorescent microalgal cells under study.

It is worthy of note that the HS-AFM technique struggles with the differential in height represented by individual empty array wells (Figure 2e), the data clearly demonstrating the well base is not in contact with the cantilever and thereby generating an untrue well depth value. Cell height offers a Z axis differential similar in scale to this array well height. To avoid a similar misrepresentation, and incorrect data collection, of sample cell Z axis dimensions, the arrays prevent the cantilever tip from traversing the full sample height. To achieve this, with sample cells embedded properly in the array, the cantilever tip interacts with only the presented, contained, topmost cell surface which is in a comparable imaging plane to the array surface.

**Figure 3:**
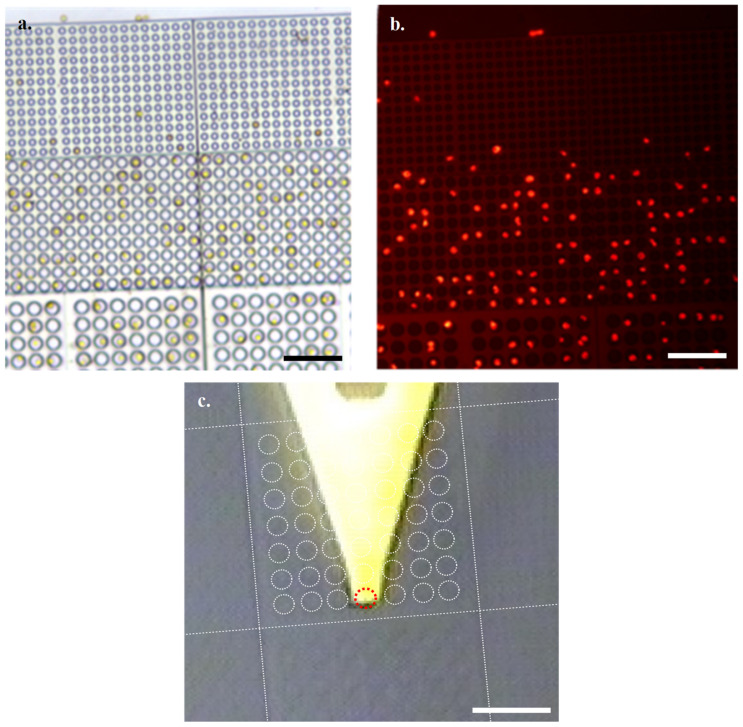
Settled *Emiliania Huxleyi* (*E. Huxleyi*) cells at ×40 obj by (**a**). optical microscopy and (**b**). fluorescence microscopy (Scale bars 50 µm) (**c**). In-line HS-AFM optical microscope of array with overlay highlighting arrangement of wells relative to cantilever (Cyl 10 block overlay, Red dashed circle marks the array well directly under the cantilever tip, Scale bar 50 µm).

Through imaging of the natural fluorescence of chlorophyll with an N2.1 filter, combined with optical microscopy, it is easy to identify suitable viable cells for subjecting to HS-AFM analysis (Figure 3a,b). Using the grid nature of the array to identify coordinates of interest, we then located cells of interest following transfer to the HS-AFM via AFM cantilever tip alignment (Figure 3c).

**Figure 4:**
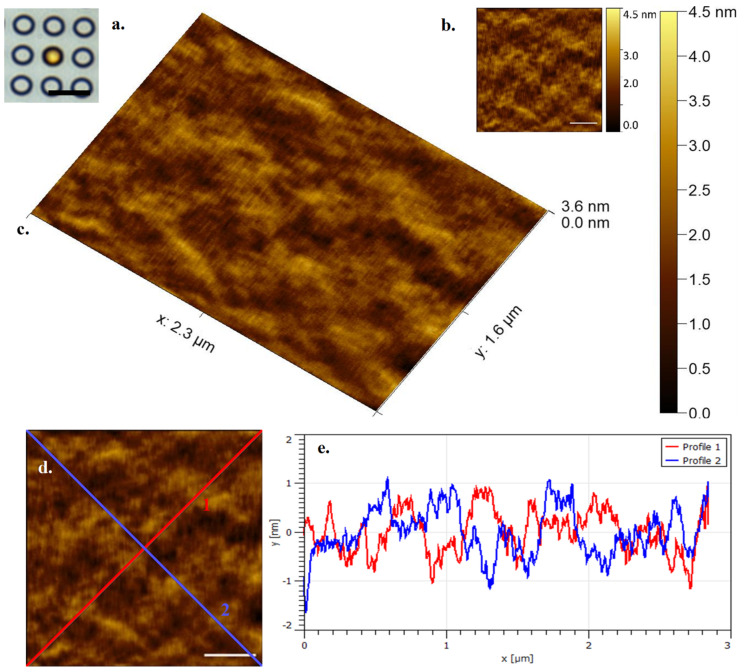
(**a**). (Inset left) *E. huxleyi* cell visualised by optical microscopy at ×63 obj (Cyl 5) (Scale bar 10 µm) (**b**). (Inset right) HS-AFM data seen in Figure 4c (Scale bar 500 nm) (**c**). *E. huxleyi* cell visualised by HS-AFM in liquid (3D render, data captured at 2 fps) (**d**). Diagonal transects (Scale bar 500 nm) (**e**) Diagonal transects marked in Figure 4d showing *E. huxleyi* surface topography.

Figure 4 shows a settled *E. huxleyi* cell in a ‘Cyl 5’ well by optical microscopy and a correlative, select, single frame of data by contact mode HS-AFM collected in 0.5 s in a physiologically relevant environment (in this case submerged in phosphate buffered saline). Membrane structure and variation can be seen that most likely represents different localised lipid compositions and associated membrane proteins. It is true that the physical constraint of the array structure might affect overall cell mechanical response, however effective sample immobilization provided by the arrays is of great importance for successful HS-AFM data collection.

**Figure 5:**
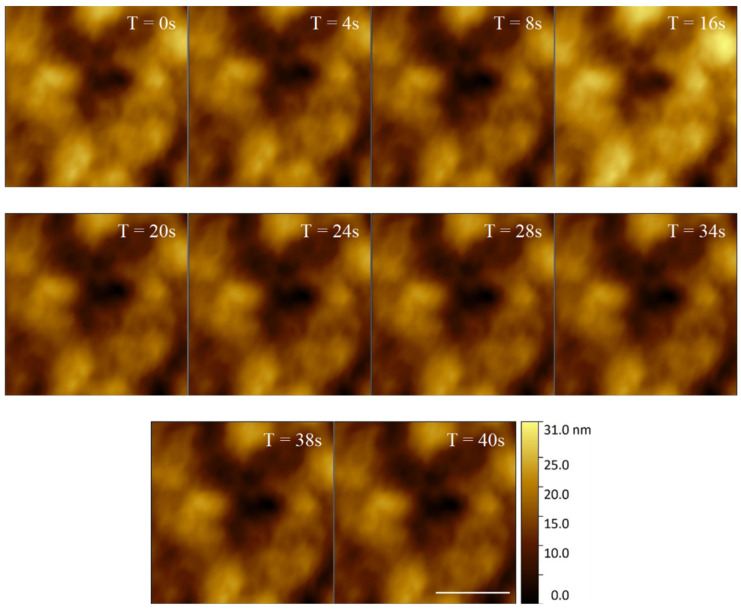
40 s time series of an *E. huxleyi* cell membrane visualised by HS-AFM in liquid (Scale bar 500 nm, Individual frames captured at 2 fps).

**Figure 6:**
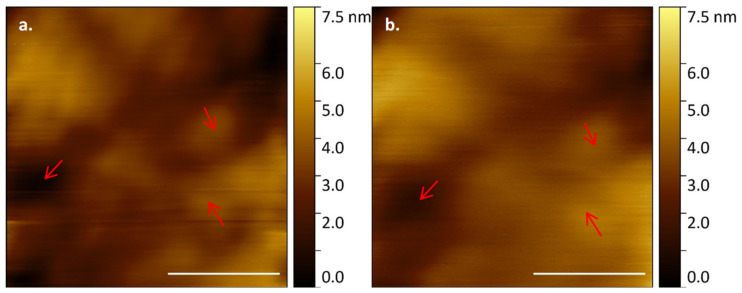
*E. huxleyi* cell membrane visualised by HS-AFM in liquid (Scale bar 500 nm, Individual frames captured at 2 fps) from near continual data acquisition at (**a**) T = 0 and (**b**) T = 5512 s; arrows mark persistant, recognisable features of the membrane.

*E. huxleyi* cell membranes were shown to have distinguishable structural features when imaged over a longer timescale (Figure 5). This structural consistency and natural fluorescence of cells can be used as a proxy for viability during longer AFM data acquisitions. Indeed, building on our visualisation of the same regions over relatively short periods of time (Figure 5), we then successfully imaged an individual cell continuously with HS-AFM for 5512 s (92% tip contact time). The membrane retained clearly identifiable features, although a drop in resolution was observed over time due to AFM cantilever tip quality degradation (Figure 6). The same microscale region could be studied for a prolonged period of time by positioning and orientation by stable structural patterns observed. Crucially, even following ~1.5 h of AFM analysis the whole cell remained optically and fluorescently viable with no obvious sign of membrane damage.

## 4. Discussion

We have demonstrated that live *E. huxleyi* cells can be observed at high temporal and spatial resolution in a liquid environment with contact mode HS-AFM. This was achieved by mechanically immobilising the cells in a 3D printed array during preparation. The algal cells were not chemically modified or fixed in any way during preparation and survived in the grids for more than 1.5 h. Due to array design and optimization flexibility, we anticipate any single cell should be suitable for immobilization with this technique. These could be mammalian, plant, fungal, etc. The technique may have limited success for smaller viruses and bacteria due to the current resolution limit of the Nanoscribe instrumentation, however this may not remain a limitation if the technology and resolution improve further.

Crucially, the arrays are chemically inert and the cells are not fixed to the grid chemically, but rely on a mechanical settling and restriction, making the arrays fully reusable and the cells potentially recoverable if necessary. Indeed, in our experiments a 70% ethanol wash followed by DI water rinse was identified as a suitably robust and reliable method of cleaning. Over time the accumulation of cellular debris is likely to occur following repeated use, but this is not likely to be an insurmountable problem with tougher cleaning regimes.

Recent developments in industrial grade (e.g., Quantum X; Nanoscribe, GmbH) 2PP printers will enable the cost reduction benefits of mass production to be realised and enable single use array analysis, which will benefit pharmaceutical applications. The screening for cell surface protein expression is a particularly promising application of this technique. Currently the material cost of a grid is estimated at £17–20 and printing run time is 15 h.

The regularity of our grid structure design allowed for easy transfer of cell location between optical, fluorescence and atomic force microscopes, critically without having all technologies in line. This makes the method applicable and adaptable to many existing instrumental setups and workflows.

Here, we have focussed on one cell type and one grid design as an example relevant to our on-going laboratory studies, however the method platform allows for high customisation in design specificity. By modifying design parameters, differences within and between arrays can be swiftly iterated to suit the sample of interest or to separate major phenotypic differences. For example, in the world of marine algal viruses (our typical area of research), un-calcified, spherical, *E. huxleyi* are optimally measured in cylindrical 5 × 5–8 × 8 µm wells, however calcified cells would be better suited to the larger 10 × 10 µm wells. A different species of algae could require an entirely different well size and/or shape. With a lower resolution limit of 0.1 µm and an upper limit of mm’s with the latest instrumentation, the potential for high sample variability applicability is present. The array loading efficacy could be used as a simple selection process. For example, haploid gametes vs. diploid cells for microalgae [21] through to selecting for cancerous human cells based on their morphology [22].

With this technique it will be possible to learn a huge amount about the structural functionality of suitable and diverse live cell membranes in their most natural state. The array fabrication method is relatively easy and versatile, with sample preparation being straight forward and fast. We are using this technique to analyse spherical algal cell samples with a high-speed contact mode atomic force microscope. There should be no limitations when using a standard or high-speed tapping mode or force spectroscopy based atomic force microscopes for analysis of samples, opening up a plethora of applications and scientific hypothesis to test. Using this array as a platform it is easily possible to immobilise numerous cells and cell types simultaneously. This provides the ability to study multiple cells at multiple locations under modifiable environmental conditions. These could be anything from how genetic modification or cancerous cells can change membrane structural functionality and properties [23], viral adhesion and infection localisation [24], mechanics studies and even response to pharmacological drugs [25]. Indeed, coupling the capability of HS-AFM as a tool, with this presented methodology could lead to preparations suitable for high throughput diagnostics and screening applications, opening up exciting opportunities for biomedical research and development pipelines.

## Figures and Tables

**Table 1:** Original block designs for designed cell array as seen in Figure 1.

Block Label (X × Y × Z Block Dimensions, Type of Pit Design, Pit Diameter)	Cylinder Diameter	Cylinder Depth	Feature
100 × 100 × 5 Cyl 5	5 µm	5 µm	Full block depth
100 × 100 × 8 Cyl 8	8 µm	8 µm	Full block depth
100 × 100 × 10 Cyl 10	10 µm	10 µm	Full block depth
100 × 100 × 5 Half Cyl 5	5 µm	2.5 µm	Half block depth
100 × 100 × 8 Half Cyl 8	8 µm	4 µm	Half block depth
100 × 100 × 10 Half Cyl 10	10 µm	5 µm	Half block depth
100 × 100 × 8 Taper 5–8	8–5 µm	8 µm	Truncated cone, diameter 8um taper to 5 µm over 8 µm Z
100 × 100 × 10 Stack 5–10	5 µm, 10 µm	5 µm, 5 µm	10 µm diameter 5 µm height cylinder stacked on 5 µm diameter 5 µm height cylinder

## Data Availability

The data presented in this study are available on reasonable request from the corresponding author.
